# Exploring different health care providers´ perceptions on the management of diarrhoea in cholera hotspots in the Democratic Republic of Congo: A qualitative content analysis

**DOI:** 10.1371/journal.pgph.0002896

**Published:** 2024-03-19

**Authors:** Mattias Schedwin, Aurélie Bisumba Furaha, Helena Hildenwall, Kelly Elimian, Espoir Bwenge Malembaka, Marc K. Yambayamba, Birger C. Forsberg, Wim Van Damme, Tobias Alfvén, Simone E. Carter, Placide Welo Okitayemba, Mala Ali Mapatano, Carina King

**Affiliations:** 1 Department of Global Public Health, Stockholm, Sweden; 2 Astrid Lindgren Children’s Hospital, Karolinska University Hospital, Stockholm, Sweden; 3 Paediatric Department, Hôpital Provincial Général de Référence de Bukavu, Bukavu, Democratic Republic of the Congo; 4 Department of Clinical Science, Karolinska Institutet, Intervention and Technology, Stockholm, Sweden; 5 Exhale Health Foundation, Abuja, Nigeria; 6 Department of Epidemiology, Johns Hopkins Bloomberg School of Public Health, Johns Hopkins University, Baltimore, Maryland, United States of America; 7 Center for Tropical Diseases and Global Health, Université Catholique de Bukavu, Bukavu, Democratic Republic of the Congo; 8 Vetsuisse Faculty, Section Epidemiology, University of Zurich, Zurich, Switzerland; 9 Department of Epidemiology and Biostatistics, Kinshasa School of Public Health, Kinshasa, Democratic Republic of the Congo; 10 Department of Public Health, Institute of Tropical Medicine, Antwerp, Belgium; 11 Sach’s Children and Youth Hospital, Stockholm, Sweden; 12 Public Health Emergencies, UNICEF, Kinshasa, Democratic Republic of Congo; 13 Programme National d’Elimination du Choléra et de Lutte Contre les Autres Maladies Diarrhéiques, Kinshasa, Democratic Republic of Congo; 14 Department of Nutrition, Kinshasa School of Public Health, Kinshasa, Democratic Republic of Congo; London School of Hygiene & Tropical Medicine, UNITED KINGDOM

## Abstract

Global cholera guidelines support wider healthcare system strengthening interventions, alongside vertical outbreak responses, to end cholera. Well-trained healthcare providers are essential for a resilient health system and can create synergies with childhood diarrhoea, which has higher mortality. We explored how the main provider groups for diarrhoea in cholera hotspots interact, decide on treatment, and reflect on possible limiting factors and opportunities to improve prevention and treatment. We conducted focus group discussions in September 2022 with different healthcare provider types in two urban and two rural cholera hotspots in the North Kivu and Tanganyika provinces in the Eastern Democratic Republic of Congo. Content analysis was used with the same coding applied to all providers. In total 15 focus group discussions with medical doctors (n = 3), nurses (n = 4), drug shop vendors (n = 4), and traditional health practitioners (n = 4) were performed. Four categories were derived from the analysis. (i) Provider dynamics: scepticism between all cadres was prominent, whilst also acknowledging the important role all provider groups have in current case management. (ii) Choice of treatment: affordability and strong caregiver demands shaped by cultural beliefs strongly affected choice. (iii) Financial consideration on access: empathy was strong, with providers finding innovative ways to create access to treatment. Concurrently, financial incentives were important, and providers asked for this to be considered when subsiding treatment. (iv) How to improve: the current cholera outbreak response approach was appreciated however there was a strong wish for broader long-term interventions targeting root causes, particularly community access to potable water. Drug shops and traditional health practitioners should be considered for inclusion in health policies for cholera and other diarrhoeal diseases. Financial incentives for the provider to improve access to low-cost treatment and investment in access to potable water should furthermore be considered.

## Introduction

An estimated 290000 children die each year before five years of age in the Democratic Republic of Congo (DRC) [1]. About 10% of these deaths are caused by diarrhoeal diseases, including cholera [2]. Cholera has the potential to cause large outbreaks, especially affecting children, with the national outbreak in the DRC in 2017 resulting in more than 53000 reported cases and 1000 deaths [3]. Cholera outbreak response is often organised vertically with dedicated treatment centres [4], and more recently using ‘case-area’ intervention approaches in which households with a reported case are specifically targeted [5]. Current global guidelines recommend wider health system strengthening to better prepare for future outbreaks [6]. Local health system strengthening for the management of disease outbreaks is also considered an effective way to gain community trust and build long-term local capacities [7].

Wider health system strengthening interventions could also concurrently improve the case management of other types of diarrhoeal diseases, which are estimated to cause more annual deaths than cholera. The estimation is 95000 deaths from cholera globally per year [8], and an estimated 480000 deaths per year due to diarrhoeal disease in children below five years of age globally [2]. The national cholera program in the DRC is mandated to not only reduce the cholera burden but also address all other diarrhoeal diseases [9].

Basic treatment for cholera and other diarrhoeal diseases in children are similar, where the cornerstone is oral rehydration solution (ORS) and zinc [10]. Access to accurate case management for diarrhoeal diseases is currently low, despite being an efficient target for lowering sub-national disparities in child mortality in the DRC [11]. For example, an estimated 11% of children under five suffering from diarrhoea received ORS and zinc in 2018, and 27% received any type of approved oral rehydration therapy [12].

Well-trained healthcare providers are essential for a resilient health system and high-quality case management. For high-quality case management of diarrhoea, including cholera, policies and interventions need to be contextualised. This study aimed to explore how the main provider groups for diarrhoea in cholera hotspots interact; how the different provider groups decide on treatment for children with diarrhoea; and how they reflect on possible limiting factors of the current situation as well as opportunities to improve prevention and treatment of diarrhoeal diseases.

**Box 1. Overview of the DRC health system**

The DRC has a pluralistic health system with formally trained providers, pharmacies and drug shops, and traditional health practitioners all providing care [13]. Pharmacies and traditional health practitioners are regulated by the Ministry of Health [14,15]. In 2017 there were an estimated 109 pharmacies, concentrated in urban settings, and 8000–10000 unregistered drug shops [16]. Drug shops are often operated by individuals without pharmaceutical training [16], and in pharmacies the licensed pharmacist is unlikely to manage customer interactions [14]. Self-medication with medicine bought over the counter from a drug shop or pharmacy is common practice in the DRC [17–19]. What can and cannot be prescribed over the counter is regulated by law, but is often not adhered to [14]. Previous studies have reported use of traditional medicine between 8% to 100% for different communities and patient groups within the DRC [20]. Plant-based traditional products are most commonly used [20], however other types of treatments such as spiritual therapies also exist [21]. Similar to the pharmaceutical market, traditional health practitioners are numerous and not well-regulated [22]. State involvement in health financing is low, and both facilities and providers rely heavily on user-fees for salary expenses as well as for procurement of equipment and medications [23]. For providers, per diems, top-up from external actors and side businesses are important income sources [23].

## Methods

### Design and timeline

We conducted focus group discussions (FGDs) in September 2022, as part of a larger mixed-method study exploring the healthcare system’s capacity to treat diarrhoeal diseases in children aged 6–59 months and participate in cholera surveillance. Case management focused particularly on children since they contribute a high morbidity and mortality burden, and since certain case management aspects differ between children and adults. As we intended to explore the healthcare system that exists in the DRC, FGDs were performed with formal providers—medical doctors and nurses, and informal providers—pharmacy and drug shop vendors (DSV) and traditional health practitioners (THP)–Box 1. Due to the low percentage of pharmacies compared to drug shops in the DRC, we use ‘*drug shop’* for both pharmacies and drug shops.

### Setting

The study was performed in the provinces of North Kivu and Tanganyika in eastern DRC. The two provinces have among the country’s highest annual incidences of cholera, with North Kivu accounting for 6451 (35%) and Tanganyika 3189 (17%) of the total 18517 suspected cholera cases reported nationally in 2022 [24,25]. Both provinces suffer complex humanitarian emergencies underpinned by armed conflict. Out-of-pocket payment for healthcare is common, however donor-subsidies are also present given the humanitarian context [26]. Humanitarian health interventions in the DRC often target epidemic prone diseases, as through cholera care subsidisation or donation of outbreak response supplies, or subsidisations for specific groups (e.g. primary care for children 0–59 months) [27]. In North Kivu, the health zone of Karisimbi, situated in the provincial capital Goma and the rural health zone of Kirotshe, about 30 kilometres outside of Goma, were selected. In Tanganyika, the study was conducted in the Kalemie and Nyemba health zones (territory of Kalemie). These two health zones together cover the provincial capital Kalemie and both extend out to rural areas. The estimated population sizes in the included health zones ranged from 380000–720000 for 2022 [28].

### Sampling

One urban and one adjacent rural setting, classified as cholera ‘hotspots’ [4], were included for each province. Rural settings near provincial capitals were selected because of their high cholera burden and accessibility for security reasons. More rural parts of the territory of Kalemie and the health zone of Kirotshe were excluded due to insecurity and limited geographical accessibility. We aimed to perform one FGD with 4–8 participants per setting for each cadre of provider: medical doctors, nurses, DSVs, and THPs (n = 16). After the sampling lists were obtained, we realised that the number of medical doctors in rural Kalemie were few and spread over large distances making the performance of an FGD unethical, since this would leave facilities without medical doctors for a long time. Therefore, no FGD was performed for medical doctors in rural Kalemie. The health facilities were identified through lists, obtained from the provincial health offices, containing information about existing public, private and confessional facilities. Drug shops were obtained the same way, except for rural Kalemie, where no such list existed, and the team conveniently recruited participants from four drug shops operating in a limited geographical zone by going physically to the zone and recruiting participants available at the time of the visit. THPs were identified with help from the provincial THP association office. No distinction was made for treatment types provided by THPs. Participants available on the day of the FGD were purposefully selected to represent as great heterogenicity as possible for the level/type of care, ownership, and geographical setting.

### Data collection

FGDs were performed separately for each cadre to minimise dominating-voice effect on participants’ views. The overall aim of the study was explained to the participants before commencement. The FGDs took place near the local health office, or in the UNICEF local office, with only moderators and participants present. Two moderators from the community with extensive previous experience performing FGDs, working for the UNICEF Integrated Analytics Cell [29], conducted each FGD (S1 Table). The FGDs followed a topic guide that was developed with the aim to capture broader perspectives of shared experiences and reasoning around diarrhoea case management, barriers and facilitators, incentives for good and bad practice, and perceptions around the current government and external actors’ approaches for diarrhoeal diseases and cholera. The topic guide was inspired by a guide developed to explore healthcare workers’ experiences and reasoning around pneumonia case management in Nigeria [30]. The guide was developed and reviewed by multiple study team members. Piloting was performed through mock FGDs with moderators acting as both participants and moderators. The topic guide was developed in French and refined and translated into Swahili (the ‘lingua franca’ of the study setting) during the group training. The final version is available in the S1 Text. The FGDs were performed in the preferred language by the participants, and participants were allowed to use both French and Swahili to express their views during the discussion. Discussions took 60–140 minutes, were recorded and transcribed verbatim, and when performed in Swahili, translated into French.

### Data analysis

All data was collected before the analysis started. We used content analysis with an inductive manifest approach, creating condensed meaning units, codes, sub-categories and categories, moving back and forth as needed [31]. However, during the process of analysis, we found some categories aligning closely to topics in the guide, and therefore took a more deductive approach to the data. Coding was performed by MS using Microsoft Word and PowerPoint. All transcripts were read, and from this, an initial codebook was developed and then applied across all transcripts before organising codes into meaning units. At this point, the categories aligned with the topic guide, so MS went back and re-organised the meaning units. Once a coherent and objective coding tree was considered in place, all transcripts were re-read to ensure that perspectives were balanced, and no important aspects had been left out. Transcripts were maintained in French, and all further steps were done in English. Quotes were translated to English by MS. A representative subset of FGDs were read by AF, and the initial analysis and interpretation were discussed between AF and MS. After some minor adjustments, both authors were in agreement on the interpretation, and this was then shared with the other authors. The same coding was applied to formal and informal providers in the analysis.

### Ethics and reporting of results

Written informed consent was obtained from all participants. Ethical approval was obtained from the ethical review board at Kinshasa School of Public Health (ESP/CE/16B/2022) and the Swedish Ethical Review Authority (Dnr 2022-02663-01). The provincial and health zone offices approved the conduct of the study. Results were reported in concordance with the consolidated criteria for reporting qualitative research checklist [32].

## Results

**Fig 1:**
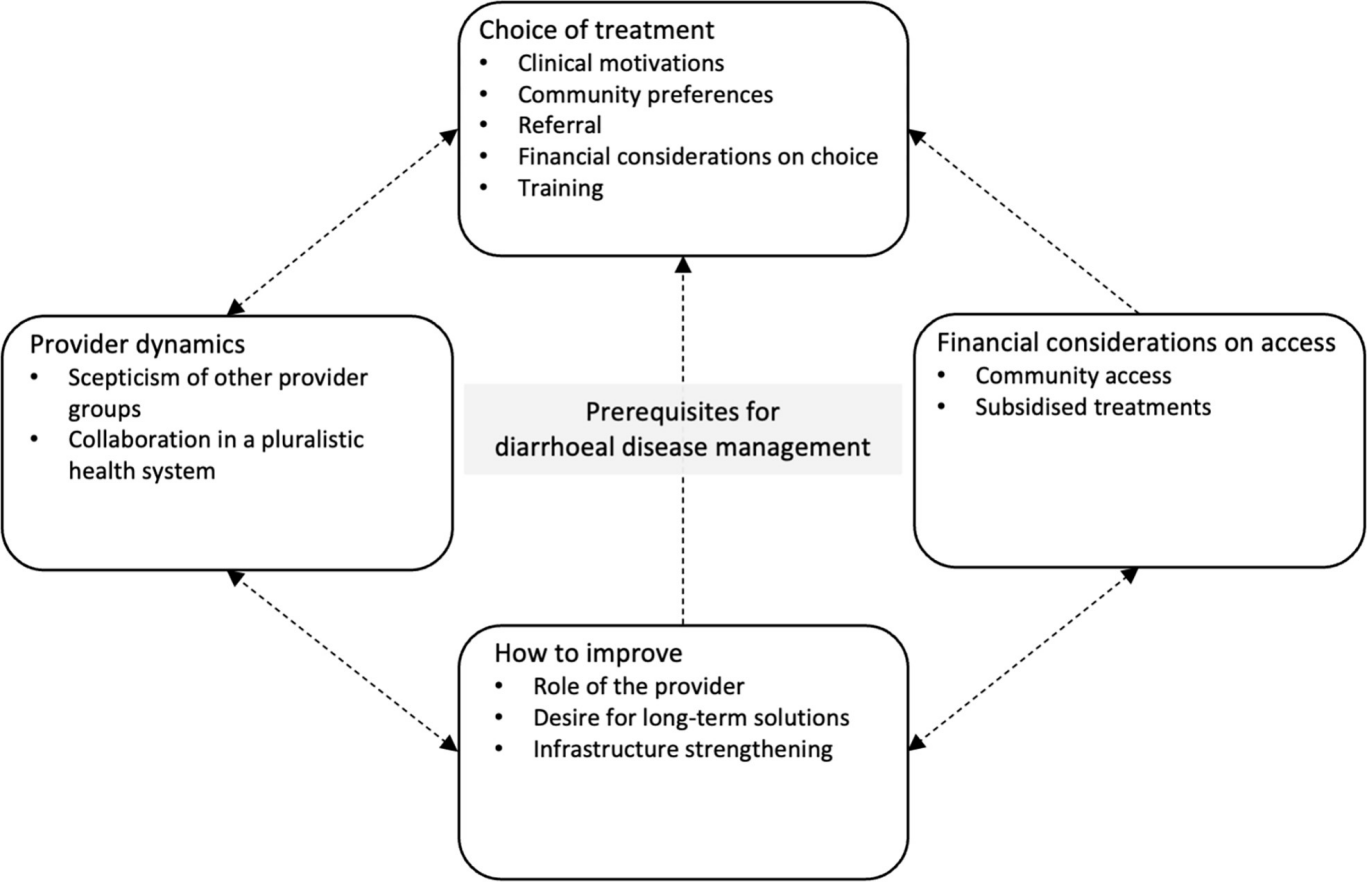
Schematic of categories and sub-categories. Arrows indicate how categories link.

A total of 15 FGDs, with 4–8 participants in each group, were conducted (Table 1). We present data under the following categories (Fig 1): provider dynamics; choice of treatment; financial considerations for diarrhoeal case management; and how to improve. We present results by sub-category within each category. Codes are in the S2 Table.

**Table 1:** Number of participants in focus group discussions by cadre.

	Focus group discussion participants (N = 84)			
	Medical doctor n = 20	Nurse n = 27	Drug shop vendor n = 24	Traditional health practitioner n = 25
**Urban**	**12**	**11**	**14**	**13**
*North Kivu*	*5*	*6*	*6*	*5*
*Tanganyika*	*7*	*5*	*8*	*8*
**Rural**	**8**	**16**	**10**	**12**
*North Kivu*	*8*	*8*	*6*	*8*
*Tanganyika*	0*	*8*	*4*	*4*

One focus group was performed per setting. * No focus group was performed for medical doctors in rural Kalemie because facilities in this area are spread over large distances and only have one or two doctors employed, making the performance of an FGD unethical since this would leave the facilities without doctors for an extended period.

### Provider dynamics

We found a sense of scepticism and a lack of mutual respect for the role and potential of the other groups providing care (health facilities, DSVs, and THPs). All three groups were aware of the plurality of providers in the system and acknowledged that this plurality affects the care they themselves provide. They were in agreement that interventions to improve case management need to include the other provider groups to be truly effective.

*‘We must not forget the traditional health practitioner sector*. *They [traditional health practitioners] are more consulted than the public facilities*, *even the private ones*. *Therefore*, *if you forget the traditional health practitioners*, *you should know that the population will [continue] suffering from diarrhoeal diseases and you will find many deaths*.*’* (Medical doctor, Kirotshe)

#### Scepticism of other provider groups

Medical doctors and nurses expressed discontent that caregivers often seek care at drug shops and THPs, delaying appropriate medical care in health facilities. This was in agreement with DSVs and THP’s own perceptions of feeling frustrated about caregivers not listening to their advice to seek care at higher-level facilities.

*‘… what prevents us from responding to cases of diarrhoea is the circuit*. *Before arriving at the hospital*, *the patient goes through traditional healers*, *pharmacies*, *etc*., *and is then taken to a hospital*.’ (Nurse, Kirothse)

Both DSVs and THPs felt unappreciated and neglected. They considered themselves to be major contributors to medical care, being more accessible, and having better community trust, especially since health facilities are too expensive to access. Medical doctors, nurses and DSVs were further clear that the common use of drug shops and THPs was due to caregiver preferences.

‘*…we drug shop vendors are the first line of care*, *and you can see that we treat many more cases of diarrhoea than the health centres*. *Because the patients start with us drug shop vendors*, *and if there is no improvement*, *it is when we refer them*, *and then they go to the health centres*.’ (DSV, Karisimbi)

*‘They first consult us*. *They explain their problems and ask for our point of view*. *The patients are not afraid to come to us because we are from the same neighbourhood*. *They go to the health centre after having gone through us*.*’* (THP, Kirotshe)

#### Collaboration in a pluralistic health system

Despite the negative perceptions, all three groups expressed the need for closer collaboration and trust between and within the provider groups to improve case management and community health. Competition between providers and within provider groups seemed to be a frustration for all provider groups.

*‘What doesn’t work is the multiplicity of facilities*, *which creates a great deal of confusion for patients*. *Patients may have a bad testimonial from someone who has been disappointed by another traditional practitioner and [now therefore] lacks confidence in my facility*’ (THP, Karismibi)

The potential benefits of collaborating within groups were brought up by a nurse who said that better collaboration between public and private providers could reduce negative events such as stockouts. Several THPs raised that biomedical and traditional medicine complement each other, asking for closer collaboration.

*‘If there is good collaboration between traditional and modern medicine*, *we can combat diarrhoeal diseases in children and reduce the death rate together*. *We have skills that the moderns do not have*, *the moderns also have skills that we traditional health practitioners do not have*.*’* (THP, Kirothse).

### Choice of treatment

#### Clinical motivations and community preferences

Several factors influenced providers’ choice of treatments, including their own orientation and applicability of guidelines, community preferences, late care seeking, and infrastructural issues such as stock-outs and diagnostic capacity. Medical doctors and nurses primarily mentioned prescribing treatment according to guidelines, and guidelines were also said to be used by some DSVs. THPs seemed more inclined to prescribe based on personal preferences, not adhering to biomedical guidelines. Prescription decisions were also said to be compromised by stockouts for both health facilities and drug shops.

‘*When you start self-medication at home*, *you [might risk] coming with the child already in need of plan C*. *We cannot find the veins*, *and while you search for the veins*, *the child dies*. *It is deplorable*.’ (Medical doctor, urban Kalemie)

‘*This protocol is like a bible for us …*’ (Nurse, Kirotshe)

Caregiver demands were important for all three provider groups, and fear was common that the caregiver would seek care elsewhere if demands were not met. Participants said it was common for caregivers to find that treatment according to the protocol (i.e. ORS and zinc) to be insufficient. Nurses said that private health facilities were more accommodating to caregiver demands than public, whilst concurrently acknowledging that accommodating these demands also occurs in public health facilities.

‘*In the private sector*, *the patient is treated as he or she wishes; it is the patient who dictates the providers*.’ (Nurse, Kirothse)

Cultural beliefs and traditions were said to be strong influencers of what treatment is demanded by the caregivers. Medical doctors, nurses and DSVs said that common misconceptions of treatment in the community are deeply rooted and maintained in part by religious leaders, prayer homes, neighbours, DSVs, and THPs. These beliefs can make it difficult to convince the caregiver to accept the recommended treatment.

’… not to think that as soon as the child has an illness it can be taken to the fetishists or to a prayer home…’ (Nurse, rural Kalemie)

‘… but also look at the traditional health practitioners, the prayer rooms. They also do damage.’ (DSV, Kirothse)

Medical doctors and nurses said that caregivers could come with direct requests to the health facility of what needs to be done, not trust the medical personnel’s suggestion, or continue traditional treatments concurrently. Nevertheless, medical doctors, nurses, and DSVs stated that they try to convince caregivers to provide the correct recommended treatment. THPs did not give any impression of being non-aligned with the demands or having to try to persuade caregivers to accept treatment.

‘*There are patients who are in the hospital but who still consult traditional health practitioners*. *You prescribe them your treatment*, *and they bring back something else*.’ (Medical doctor, Kirotshe)

*‘As for the drug shop vendors*… *some of them often mix several medicines*, *tetracycline*, *amoxicillin*, *chloramphenicol*, *rifampicin*… *Someone showed me that he had been given all these medicines and asked me if I also could prescribe him these same medicines*. *I was scared*.*’* (DSV, Kirotshe)

#### Referral

All provider groups reported referring children with diarrhoea, and there was agreement that decisions around referral were challenging. However, providers’ motivations for referral and timings differed. A key consideration among all providers was the cost of care and, therefore, the decision to refer to subsidised facilities, but this had to be balanced against distance and the possibility of a child dying during the onward journey. This was accompanied by the reported frustration that feedback was rarely provided after referral, which meant that opportunities to learn whether or not their decision was justified were missed.

‘*You take on the patient*, *you help him*, *you do what you have to do*, *but at a certain point it’s going to weigh on your shoulders*. *He is not going to pay*, *and we still have to keep rehydrating him*. *We have facilities where it’s free*, *so we refer him there*, *provided he can get other care elsewhere where [his inability to pay] is tolerated better*. *So*, *it just depends on the means and the limits*. *It is in that sense*.*’* (Medical doctor, Karismbi)

All providers, including THPs, reported similar criteria for deeming a case in need of referral (dehydration status, treatment failure), but there were tensions around timings of onward referral. One THP stated that traditional medicine takes a longer time to have effect than modern medicine, and THPs reported both waiting days before referral and urgent referral practices for severe cases. Doctors and nurses also mentioned referring children to prevent medication stock-outs, which was not raised by other providers.

*‘For diarrhoea*, *if the child can no longer stand up and keeps wanting to go to bed*. *You say to yourself*, *this is a case that needs to go to the hospital*.*’* (THP, rural Kalemie)

#### Financial considerations on choice

Across groups, the type of treatment provided was influenced by the financial capabilities of the caregiver. Low-cost drugs of lower quality were offered to caregivers with low capability to pay, and more expensive treatments were offered to caregivers with higher capability. Participants also stated that caregivers with higher payment capability expected to be offered more expensive treatments and could be offended when suggesting low-cost treatment recommended by guidelines. Furthermore, these caregivers were seen as an opportunity to increase revenue for the facility. All provider groups acknowledge the existence of providers within their own cadre that prescribe based primarily on their own financial gains and not patients’ needs. Even hospitalisation was said to occur for the same reason.

*‘There is a principle in medicine*, *“the rich pay for the poor”*. *You can’t tax a poor person like a rich person*, *so the rich have to compensate for the poor*.*’* (Medical doctor, Karisimbi)

For health facilities, overprescription of drugs was mentioned to occur due to lack of laboratory diagnostic methods or the caregiver not being able to pay for the laboratory tests, thereby prescribing treatments on an empirical basis. DSVs said that what they keep in stock is mainly based on what medication the population can afford and the type of medication the community demands, as well as those recommended by guidelines.

‘*We add antibiotics even though it’s not recommended because*, *quite simply*, *we don’t have a lab to find out what’s causing the diarrhoea*.’ (Medical doctor, Kirothse)

#### Training

All provider groups considered that knowledge within their cadre about case management was insufficient and asked for improvement opportunities ranging from external training programs, in-house training, and sensitisation campaigns. Both medical doctors and nurses expressed that it is common that personnel providing continuous care do not have the necessary skills to provide care nor know when to ask for help, a perspective also brought up for cholera treatment centres.

‘*To continue to develop our knowledge of the management of diarrhoeal diseases*, *because ignorance can also lead to treatment being omitted or misdirected*. *But by educating ourselves and participating in capacity-building courses … we can be more efficient in managing these diseases*.*’* (DSV, urban Kalemie)

Medical doctors and nurses expressed that DSVs and THPs need to be trained in proper case management. DSVs and THPs correspondingly asked for training to be able to collaborate with health facilities and provide first-line care. However, for THPs themselves, the superiority of traditional treatment was more prominent than the demand for biomedical training.

‘*So*, *I don’t know if you can help us to identify these traditional health practitioners and drug shop vendors*, *[and] the private structures so that they are trained in the management of diarrhoea and so that we have a common language*.*’* (Nurse, Kirothse)

### Financial considerations on access

#### Community access

Financial aspects were important among all provider groups. However, empathy was simultaneously strong with providers trying to find solutions to give patients access to treatment. Providers reported the following solutions for when caregivers cannot pay: reducing price, giving credit, pooling of cost, referral, subsidisation by the provider, offering part of treatment, providing lower quality drugs, leaving patient untreated, and providing treatment free of charge. These, except the two latter, also concurrently provide financially for the provider and avoid having to close the practice due to financial costs. All providers expressed that poverty needs to be mitigated to facilitate access to care. Lower costs were, according to all three groups, a main reason why care is sought first at THPs or drug shops and seeking care late or not at all in health facilities.

‘*You will find that parents put a lot of trust in their drug shop vendors … you see that the case requires going to hospital*, *but the parent persists in buying the medicines rather than going to hospital because of lack of means*, *and there are some parents who lose their children as a result of putting too much trust in the drug shop vendor*.’ (DSV, urban Kalemie)

#### Subsidised treatment

All provider groups were clear that subsidised or free treatments provided by external actors need to consider economic gains for the provider if they are to be effective. Insufficient or no pay was mentioned as contributing to a lack of motivation and distract from providing optimal care. For drug shops, this could additionally compromise their existence through income loss due to the subsidised treatment competing with sales of other drugs. Providers stated being less motivated to treat cholera since treatment is free of charge; prescribing additional treatment when ORS plus zinc was free of charge; and community healthcare workers not performing their tasks due to not being paid. Additionally, providing treatment completely free of charge might make the product less valued in the community. As an example, a DSV brought up a situation when condoms had been provided free of charge, and they were found lying on the street since people knew they could get new ones. Conversely, the positive aspect of attracting more customers if offering free treatment was brought up, and some DSVs said that they had no issue in providing treatment without personal gain.

‘*You as doctors need to live*, *not only save lives*, *but to create income*, *there are these two aspects*: *the humanitarian aspect and the income aspect*.’ *(*Medical doctor, Kirotshe*)*

‘*If the NGOs helped us with the oral rehydration solution it would be very helpful for us because we can [then] lower the price*.’ (DSV, urban Kalemie)

Specifically, it was mentioned that short-term subsidisation programs could have severe negative consequences for the provider. When subsidies have been withdrawn, the facility risks financial hardship/closure as caregivers will seek care elsewhere. The argument for this being that caregivers have changed what they can expect from the facility, and when this is removed caregivers go elsewhere.

‘*They [external actors] come for 3 months*, *and then they disappear again*, *only to return after 10 years*. *It makes life so difficult for us*. *Helping is all well and good*, *but you have to provide continuous support*. *That’s what I can say about this because I’ve worked in several facilities*, *and it was the same thing*: *free of charge*, *free of charge … they come*, *they pay everyone*, *they take care of everyone*, *everyone feels good*, *the doctor has his salary*. *It is good*, *things happen*. *A year later*, *they leave*, *and there are no patients coming to the facility anymore*. *You see*, *with policies like that*, *you’re helping but while destroying*.’ (Medical doctor, Karisimbi)

### How to improve

#### Role of the provider

It was raised across groups that providers need to take responsibility and be held accountable for the treatment they provide. Furthermore, all provider groups acknowledge the importance of them educating caregivers and the wider community in prevention and initial treatment.

‘*The health zone must encourage us to work together with them*. *When we treat patients*, *we have to report to the health zone*, *and in turn they have to evaluate our work against the reports we produce*, *but when we work knowing that there is no one who will ask for the report often we work sloppily* [French word ‘légèreté].’ (THP, Kirothse)

All provider groups considered themselves to have important roles during a cholera outbreak.

Medical doctors, nurses, and DSVs were all considering their role during a cholera outbreak to assist with sensitisation and refer suspected cases. Medical doctors and nurses said their primary role was to assist with case management, and alerting authorities was mentioned as another important task. DSVs also mentioned preventive measures such as providing chlorine tablets and stocking medication from the local depot when they noticed an outbreak was starting. The latter also made them function as a buffer for health facilities. THPs mentioned helping to coordinate the community during outbreaks, whilst some also acknowledged treating cholera cases independently and referring cases. All provider groups expressed wanting to be given the tools to be better and more involved during cholera outbreaks. They argued that their closeness to the community makes them efficient in informing, educating, identifying, and referring cases.

*‘This is how the epidemic is going to unfold*, *and if we receive two or three cases*, *we go straight to the pharmaceutical depot to collect medicines to facilitate the treatment of patients*.*’* (DSV, rural Kalemie)

#### Desire for long-term solutions

Providers asked for long-term support rather than short-term aid. All provider groups were aware of existing external cholera interventions and agreed that support from external actors made a big difference during outbreaks. However, at the same time, frustration was noticed concerning the short duration of outbreak interventions and the focus on putting out fires rather than working on sustainable preventive and measures. Childhood diarrhoea was said to be an ‘everyday’ disease and, therefore, does not receive the same attention as cholera, even though it causes suffering and death. Providers wished that interventions should not only occur during epidemics.

‘*But what saddens me about their approach is that they intervene when there is a case*, *whereas they should be focusing on prevention activities even when there is no case of cholera*.’ (Nurse, Kirothse)

‘*The organisations must*, *in the first place*, *I believe*, *accompany us*. *Not to help us too much*. *Why*, *because there is this problem when organisations come*, *they create what we call a dependency*, *interestingly the organisation comes to destroy what people could already do*.’ (Medical doctor, Karismbi)

All groups asked for continued support from NGOs and the government. A strong wish was raised, across provider groups, for the government to take on a bigger responsibility due to the unpredictability of NGOs and the modest efforts in combatting the drivers of the epidemics and diarrhoeal diseases. The government was asked to preferably take on the responsibility of coordinating efforts, not only for NGO and governmental interventions but also to coordinate and integrate the different provider groups. The health zone’s role in coordinating providers and external actors was specifically highlighted. Concurrently, a lack of trust in the government taking on the necessary responsibility was brought up. THPs asked for better recognition and inclusion of their cadre with additional support ranging from access to certification of their drugs to assisting with building centres where THPs can work together. Several THPs also asked to separate THPs from the Ministry of Health.

‘*I think that if we reason on the basis of responsibility*, *it is the government’s* [responsibility], *but given the shortcomings that our government has already shown*, *I prefer the NGOs to the government*. *Because the government is not for today*. *I don’t think our Congolese government can make it work*. *It is not today that it [the government] is going to solve the problems*. *It is better for NGOs to get involved in managing diarrhoea*.’ (Medical doctor, urban Kalemie)

‘*Traditional health practitioners are not recognised and do not have the confidence of the Congolese health system when it comes to treating illnesses such as diarrhoea*. *We will not receive any instructions on how to treat diarrhoea because we are not involved*.’ (Traditional health practitioner, Karisimbi)

#### Infrastructure strengthening

Availability of medication, preventing stock-outs, recurrent training in case management, educating the community, access to improved toilets and safe drinking water, and access to care in health facilities were mentioned by providers as key focus areas. Preventive measures were overall considered more important than support with curative service. Access to water sanitation and hygiene measures for the community stood out as the most wished-for intervention for all provider groups.

‘*The way we want it dealt with is to tackle the source in the first place*. *As long as we don’t address the root cause*, *I believe that diarrhoeal diseases will remain*. *So*, *we’d prefer these people to dig wells*, *which are conveniently located in the community*, *so that people can access clean water*.’ (Nurse, rural Kalemie)

## Discussion

In this qualitative study, we found important aspects of how different groups of providers in a cholera-endemic region of the DRC perceive each other, interact, and decide on treatment for diarrhoeal illness. We demonstrated their interconnectedness and how they all influence the case management of diarrhoeal diseases in children. Affordability, strong caregiver demands shaped by cultural beliefs, and financial incentives for the providers were important contributors to what treatments are received. Furthermore, we found that providers appreciated the current cholera response but asked for long-term support for basic public health measures.

We found support for including drug shops and THPs in policies for diarrhoeal disease case management, including cholera surveillance and outbreak response. Medical doctors and nurses believed that they will never be able to ensure good quality case management of diarrhoeal diseases if treatment by drug shops and THPs is not considered in public health policies. Previous research has found that health facility personnel want collaboration, but mainly through one-way referrals from THPs to health facility personnel [33]. Our results indicate a more nuanced reasoning. DSVs and THPs believed that they are important given health facilities remain inaccessible due to high financial costs. The preference for using drug shops by communities has previously been explained by lower costs, limited access to healthcare facilities, and easy access without the necessity of a prescription [34,35], which is in line with our results. For the involvement of drug shops, the Accredited Drug Dispensing Outlets approach first rolled out in Tanzania could be considered [36]. This approach aims to improve pharmaceutical services and increase access to specific medications that normally require a prescription by a trained healthcare provider. It includes comprehensive training, record-keeping, and thorough monitoring and evaluation. However, its implementation has demonstrated varying results and includes problems in supervision and maintaining trained personnel due to staff turnover [36,37]. Research from Uganda has shown that diarrhoea case management can be improved in private drug shops through training in integrated community case management; however, the authors also raise general concerns related to financing and the motivation of providers [38]. Another intervention study demonstrated that it was possible to use pharmacy sales of oral rehydration solutions to predict cholera outbreaks [39], but active reporting and follow up of sales seems to be costly and resource-intensive.

Important explanators for the use of THPs are comprehensiveness (treating both biomedical and traditional conditions), effectiveness, speed of healing, and low cost [40–43]. If THPs were to be included in policies, individuals who are dependable, open to collaboration and evaluation, and that both the community and the biomedical providers consider competent should be targeted [44–46]. Research outside the DRC has shown that some THPs are open to adopting biomedical approaches [47], and in our study, we had individuals who were open to doing so and those who seemed less likely. Important to note is that traditional treatments sometimes include potentially harmful practices and products [21]. Closer collaboration between biomedical providers and THPs could possibly attenuate the use of harmful non-evidence-based practices, create access to primary care, and have support from the WHO and the 2019 United Nations General Assembly resolution on universal health coverage [21,48]. A recent cluster-randomised trial showed that the use of traditional healers for HIV-testing increased testing [49]. A randomised control trial in India evaluating a training programme of informal providers found improved clinical skills; however, it found no improvement in prescription practices [50]. The randomised trial only included informal providers providing modern (allopathic) care, and not THPs, which one can hypothesise would be a more challenging group to influence to adopt allopathic guidelines. Furthermore, the potential gains will not be possible without the existence of genuinely pursued legal frameworks, combined with close supervision, to ensure that everyone is acting within their jurisdiction [42].

Research from Australia demonstrated that regulation of traditional medicine improved safety [51]. Given that the DRC has an insufficient health budget, adding a layer of regulation may be challenging. Insufficient supervision from underfinanced provincial health offices, unregulated establishment of private providers, a poorly regulated drug dispensing market, poor supervision of THPs, and insufficient internal control and follow-up to improve the quality of care are existing challenges for the DRC [13,14,22]. Without succeeding with the steps above, there is a risk of legitimising unsafe practices. More focus is needed from stakeholders on developing solutions for how to improve these shortcomings.

Our findings highlight the importance of affordability and community perceptions to drive caregiver demand. Access to correct treatment without suffering financial hardship needs to be ensured. DRC’s commitment to universal health coverage is a good initial step towards this [13]. This study raises important aspects to consider when designing interventions. First, financial subsidies for treatment need to consider the potential negative side effects of not including financial incentives for the providers. Currently, many providers in public health facilities are not being paid a regular salary [13], and strikes over pay occur frequently [52]. DSVs also brought up that providing treatment free of charge could jeopardise the survival of the facility. The feasibility of nudging providers through some kind of financial benefit could be considered to increase the prescription of recommended treatment. Given the diversity of providers in the DRC, upstream subsidies are more likely to be cost-effective than downstream subsidies. Subsidising the importer or producer of ORS and zinc in combination with free delivery and a fixed price for the community could, for example, be considered. This should preferably be tested small scale and be evaluated to ensure it has the desirable effect. Additionally, previous literature has demonstrated that multidimensional systems thinking is necessary when subsidisation interventions are implemented if access to high-quality care is to be realised [53]. There among mitigating demotivation of health staff due to loss of informal payments, and concurrently assuring rational diagnostic procedures and rational prescription of treatments [53]. Second, short-term subsidies risk creating negative economic side effects for the facility once the subsidies are lifted due to changing caregivers’ expectations and demands. More long-term sustainable approaches, or at least including exit strategies that mitigate potential side effects in intervention planning, should be considered. Countries that have succeeded in increasing coverage of ORS and zinc have used long-term solutions to keep costs down in both the public and private sectors [54]. Third, DSVs consistently said they keep what the community demands in stock and both DSVs and THP were said to influence community demands. Therefore, including these groups in cholera and diarrhoeal disease policies could potentially be beneficial to improve community practices.

Providers’ perceptions on what needs to be done to prevent children from falling ill from diarrhoeal diseases and getting access to correct treatment were in line with the Global Task Force on Cholera Control recommendations [6]. Focus on preventive measures, especially access to safe drinking water and sanitation, in the community was more important for the providers than curative services. Vertical short-term approaches are still necessary for emergency situations, which also had clear support from providers, especially when an outbreak occurs in a setting such as a displacement camp which is common in the DRC [55]. However, thorough long-term multisectoral work, ranging from improving access to safe drinking water, changing caregiver demands, promoting access to health care, ensuring availability of medication, as well as transparent and efficient governance that can coordinate actors is necessary to sustainably lower diarrhoeal disease burden and to decrease frequency and severity of outbreaks in cholera hotspots in eastern DRC.

This study had three key limitations. First, the perspectives of religious leaders, the community, the government, and the external actors were not included. These are all important for a complete stakeholder analysis. Second, due to the composition of the FGD groups, competition and tensions within provider groups might have been underreported. Several authors are aware of friction and competition between health facilities that can, for example, delay or prevent referral. Third, moderators were employed by UNICEF, who engage actively in cholera and diarrhoeal diseases response in the settings where the research was conducted and may have resulted in social desirability bias [56]. Interviewers were clear about the anonymity of study participants as well as the importance of obtaining truthful answers to questions to be able to inform future interventions. Perspectives identified in this study may be relevant to reflect on also for other cholera hotspots, given vertical programs to limit cholera are still the main approach, and cholera outbreaks often occur in fragile settings where pluralistic health systems are common.

## Conclusion

We found support for the idea that all provider groups should be taken into consideration when developing policies to combat cholera and lower the diarrhoeal disease burden. Medical doctors, nurses, drug shop vendors, and traditional health practitioners are interconnected, and involved in providing care for diarrhoeal diseases. Financial incentives for the provider and a low price for the community were found important to increase prescription, promotion, and use of recommended treatment. Long-term financial subsidies for treatment could be of value to increase access to treatment and should consider incentives for the provider and consider exit strategies that mitigate side effects when support is withdrawn. Focusing on preventive measures in a sustainable way with priority given to investments in access to safe drinking water had broad support among providers and higher priority than investments in curative services.

## Supporting information

S1 Checklist Inclusivity in global research. (DOCX)

S1 Table Personal characteristics of data collectors. (DOCX)

S2 Table Derived categories and codes from analysis. (DOCX)

S1 Text Focus group discussion guide. (DOCX)
