# Supplementary material for: Exploring different health care providers´ perceptions on the management of diarrhoea in cholera hotspots in the Democratic Republic of Congo: A qualitative content analysis
Source: PLOS Glob Public Health. 2024 Mar 19;4(3):e0002896. doi: 10.1371/journal.pgph.0002896 (PMC10950234; doi:10.1371/journal.pgph.0002896)
Supplement: S1 Table — (DOCX) [file pgph.0002896.s002.docx]

**S1 Table. Personal characteristics of data collectors.**

| Province | Sex | Basic training | Previous experience | Occupation at the time of the study |
| --- | --- | --- | --- | --- |
| Nord Kivu | M | Demography | Several qualitative and quantitative studies carried out with the UNICEF Integrated Analytics Cell as a researcher. | Researcher in the qualitative and quantitative data collection team at the Integrated Analytics Cell (North Kivu). |
|  | F | Psychology |  |  |
|  | F | Sales and Administration  Diploma in Community Development |  |  |
|  | M | Health sociology and anthropology |  |  |
|  | M | Teaching  Diploma in Health Community and Development |  |  |
|  | F | Social sciences  Diploma in Community Development  Diploma in Health Project Management and Administration |  |  |
|  | M | Social sciences  Diploma in Community Development  Diploma in Health Project Management and Administration |  |  |
|  | F | Nursing  Diploma in Health Community and Development |  |  |
|  | M | Agricultural and environmental sciences. |  |  |
| Tanganyika | M | Economy |  |  |
|  | M | Economy |  |  |
|  | F | Economy and labour law |  |  |
|  | F | Business Informatics |  |  |
|  | M | Nursing |  |  |
|  | F | Communication |  |  |
|  | M | Business Informatics |  |  |
|  | F | Public Health |  |  |
| *No relationship was established between researchers and participants before commencement* | | | | |
