# Supplementary material for: Exploring different health care providers´ perceptions on the management of diarrhoea in cholera hotspots in the Democratic Republic of Congo: A qualitative content analysis
Source: PLOS Glob Public Health. 2024 Mar 19;4(3):e0002896. doi: 10.1371/journal.pgph.0002896 (PMC10950234; doi:10.1371/journal.pgph.0002896)
Supplement: S2 Table — (DOCX) [file pgph.0002896.s003.docx]

**S2 Table. Derived categories and codes from analysis.**

| Categories | Sub-categories | Codes |
| --- | --- | --- |
| Provider dynamics | Scepticism of other providers groups | Sub-performance from other provider groups  What we do better  Caregivers |
|  | Collaboration in a pluralistic system | Concurrence  Collaboration  Clear roles |
| Choice of treatment | Clinical motivations | Clinical signs  Following protocol  Seeking care late |
|  | Community preferences | Caregivers influence  Imposing perceived correct treatment  Provider shortcomings |
|  | Referral | Medical reasons for referral  Non-medical reasons for referral  Reasons not to refer  Feedback on referral |
|  | Financial considerations on choice | Caregiver ability to pay  Availability of products  Capability to pay decides stock  Prescribe for maximal financial gain |
|  | Training | Providers need training  Also CTCs need training  We only get theoretical support |
| Financial considerations on access | Community access | Possibility to pay decides access  Compromising treatment due to inability to pay  Inability to pay puts us in difficulty  Solutions to inability to pay  Empathetic values guide |
|  | Subsidised treatments | Money as motivation  Need financial gain to provide subsidised treatment  Will provide donated treatment for free  Free treatment less valued  Doing good is good for business |
| How to improve | Role of the provider | Improve case management  Hold accountable and provide according to standards  Correct treatment in stock  Work with the community  Role during outbreak |
|  | Desire for long-term solutions | Long term approach  Prevention is key  Do not forget the ‘everyday killers’  Government to take responsibility  Government not reliable  NGOs support during outbreaks  NGOs are short sighted and can disturb ecosystems  Collaboration on all levels including governance  Give us traditional healers more influence |
|  | Infrastructure strengthening | WASH  Equipment and medication  Support private alternatives  Pay healthcare providers  Improve access |
