# Supplementary material for: Exploring different health care providers´ perceptions on the management of diarrhoea in cholera hotspots in the Democratic Republic of Congo: A qualitative content analysis
Source: PLOS Glob Public Health. 2024 Mar 19;4(3):e0002896. doi: 10.1371/journal.pgph.0002896 (PMC10950234; doi:10.1371/journal.pgph.0002896)
Supplement: S1 Text — (DOCX) [file pgph.0002896.s004.docx]

**S1 Text. Focus group discussion guide.**

**Group discussion guide**

**(doctors/nurses/drug shop vendors/traditional health practitioners)**

**Not considered in this study.*

1. *Welcome to the participants*
2. *Informed consent*
   - Ensure that everyone is aware of the audio recording
   - Assign random numbers to participants and reiterate confidentiality
   - Filling in the participant information sheet
3. *Introductory question (don't stay too long on this question)*
   - Can you tell me what kind of sick children you normally see in your environment?
     - Probe: How many children do you see? How many have diarrhoea?
4. *Paediatric diarrhoea*
   - What do you know about diarrhoea in children?
     - Probes:
       1. Cause
       2. Presentation
       3. Main groups affected

*(INFORMATION FOR THE INTERVIEWER: MAKE SURE THAT ALL PARTICIPANTS UNDERSTAND WHAT WE MEAN BY DIARRHOEA: LOOSE STOOLS (WITH OR WITHOUT VOMITING))*

- - Can you describe a typical case of diarrhoea in your setting?
  - How do you normally diagnose a child with diarrhoea?
    - Probes:
      1. Specific questions asked?
      2. Specific clinical signs sought?
      3. Tools used (guidelines)?
  - How do you decide if it is a severe or non-severe case of diarrhoea?*
    - Probe:
      1. Does malnutrition change the assessment?
  - How do you assess whether the child is dehydrated?*
    - Probes:
      1. Specific questions asked?
      2. Specific clinical signs sought?
      3. Tools used (guidelines)?
  - Can you describe how you treat a child with diarrhoea?*
    - Probes:
      1. Drug used and when
      2. Fluids administered - when, how and what type
      3. When to use intravenous fluids
      4. Things not to give to children (e.g. medication, types of food etc.)
      5. Does malnutrition change the way we manage?
  - Are there times when you deviate from what you consider to be proper care? *(Here we want to know about limiting factors (e.g. lack of medication, parents cannot pay). We are not looking for reasons for incorrect treatment)*
    - Probes :
      1. In what way?
      2. What are the reasons for this?
  - How do economic incentives influence the type of medicines you recommend for treating diarrhoea in children?
    - If you get free treatment from an NGO for example. Would this make you more or less likely to prescribe the drug?

1. *Economic incentives and access to medicines* [**DRUG SHOP VENDORS ONLY**].
   - How do you decide what type of medicine you have in stock to treat diarrhoea in children?
   - What would increase your tendency to prescribe Oral Rehydration Solution?
2. *Policy recommendations*
   - What are the main drivers and barriers in your efforts to address diarrhoea in children?
     - Probe :
       1. Facilitators (what works well)
       2. Obstacles (what doesn't work well)
   - What could be done in your facility to improve the management of diarrhoea in children?
   - What could be done by the community to improve the management of diarrhoea in children?
   - What could be done by the government to improve the management of diarrhoea in children?
   - What could be done by the external actors such as NGOs to improve the management of diarrhoea in children?
   - If you could choose one of these interventions (referring to the ones suggested), which would you prefer? Why or why not?
3. *Response to cholera*
   - Are you aware of any cholera interventions in the area where you work?
     - If so, can you describe them? When did they take place? By whom?
       1. How do you think these interventions were organised?
       2. Are there things you wish had been done differently?
     - If not, why do you think there are no initiatives in your area?
4. *Reflecting on one's own role in the protection, prevention and treatment of diarrhoea and in the fight against cholera.*
   - How do you see your role in protecting children from diarrhoea?
     - Probe:
       - Preventing children from getting sick
       - Home/institutional treatment
       - Knowledge of caregivers
   - How do you see your role in a cholera outbreak?
     - Do you think your role could be more effective during a cholera outbreak, and if so how?
5. *Perception of formal health care [ONLY TRADITIONAL HEALTH PRACTITIONERS]*
   - How do you see your role in treating children with diarrhoeal diseases in relation to the wider health care system (health centres, hospitals, etc.)?
   - How would you feel about working with the wider health care system to treat children with diarrhoeal diseases?
     - How would such collaboration ideally be implemented?
6. *Additional aspects*
   - Is there anything else about diarrhoea in children and what we have discussed today that we have not mentioned that you would like to add?

**Concluding question**

- Of all the things we have discussed today, what do you think are the most important issues?

**Conclusion**

- Thank you for your participation. This discussion has been very enriching.
- Your views will be a valuable asset to the study.
- We hope you found the discussion interesting.
- If you are not satisfied with something or wish to complain, please contact the local PI or speak to me later.

I would like to remind you that all of you and all of your views will be anonymous in the report.
